# Factors associated with osteoporosis in older adult women treated at a referral center

**DOI:** 10.1590/1980-220X-REEUSP-2025-0489en

**Published:** 2026-06-01

**Authors:** Maria Suzana Marques, Luiza Almeida Perdigão, Thomaz de Figueiredo Braga Colares, Ely Carlos Pereira de Jesus, Luciana Colares Maia, Antônio Prates Caldeira

**Affiliations:** 1 Universidade Estadual de Montes Claros, Montes Claros, MG, Brazil.

**Keywords:** Osteoporosis, Aged, Risk Factors.

## Abstract

**Objective:**

To analyze factors associated with osteoporosis in older adult women treated at a health care center for the older adult in northern Minas Gerais.

**Method:**

This is a cross-sectional and analytical study involving 647 women aged 60 years or older who underwent bone densitometry between 2015 and 2024. Data were collected from medical records, including sociodemographic, clinical, and lifestyle information. Osteoporosis was diagnosed using a T-score ≤ −2.5. Bivariate analyses and binary logistic regression were performed.

**Results:**

The results indicated a prevalence of osteoporosis of 46.4% among the participants. Multivariate analysis revealed that age equal to or greater than 80 years (OR = 1.60; 95% CI: 1.14–2.24) and body mass index (BMI) less than 27 kg/m^2^ (OR = 1.97; 95% CI: 1.42–2.74) are factors associated with osteoporosis.

**Conclusion:**

These findings demonstrate the high prevalence of osteoporosis in the study population, as well as the need to implement preventive strategies and interventions, especially in older adult women with low body weight.

## INTRODUCTION

Osteoporosis is the main osteometabolic disease in older adults. It is characterized by reduced bone mineral density with significant impairment of bone microarchitecture, leading to an increased risk of falls and fractures^(1,2)^. It is a condition with high prevalence worldwide, especially in European and African countries. A meta-analysis study shows that the global prevalence of osteoporosis is 18.3% (95% CI 16.2–20.7), being higher among women, reaching rates of 23.1% (95% CI 19.8–26.9)^(3)^.

The accelerated aging process of the Brazilian population^(4)^points to a growing number of older adult women demanding health services, which demonstrates the relevance of osteoporosis as an imminent public health problem. It should be noted that osteoporosis has no obvious clinical manifestations until the person suffers a fracture. For older adult, fractures are associated with increased morbidity and mortality and lead to a significant impairment in quality of life, both for the patients themselves and for their families, who have to deal with greater limitations^(2)^. This fact highlights the importance of early diagnosis of this disease, based on timely assessment of bone mineral density, and early treatment, before fractures occur^(5)^. National studies, although infrequent, highlight the relevance of the topic^(6,7)^.

One limiting factor for studies on osteoporosis concerns patient allocation. Population-based studies are difficult to conduct and involve higher costs. In this sense, some studies address prevalence and associated factors based on the population undergoing bone densitometry (BD) testing, considered the gold standard for diagnosing the disease^(8,9)^. In this context, health care centers for the older adult play an important role in the timely identification and management of osteoporosis. Throughout the northern region of the state of Minas Gerais, a single health care center for the older adult is responsible for performing BD tests through the Unified Health System (SUS). This center serves the northern macro-region of Minas Gerais, which covers 86 municipalities and a population of over 255,000 older adult^(10,11)^.

Considering that in recent years, efforts have been made to expand knowledge of the epidemiology of osteoporosis in Brazil in order to improve the effectiveness of disease prevention, and that there is still little research on the subject in the macro-region of Montes Claros – Minas Gerais^(12)^, this study aimed to analyze the factors associated with osteoporosis in older adult women treated at a health care center for the older adult in northern Minas Gerais.

## METHOD

### Study Design

This is a cross-sectional and analytical study.

### Location, Population, and Selection Criteria

The population of older adult women referred by Family Health Strategy (FHS) teams to the Reference Center for Health Care for the Elderly (CRASI) for BD tests was evaluated. This center is linked to the Clemente de Faria University Hospital and provides health care exclusively through the SUS. The service consists of a team of professionals from different areas and backgrounds, providing interprofessional health care. CRASI serves approximately 1,000 people per month and performs about 400 BD tests, covering patients referred by Primary Care teams from municipalities in northern Minas Gerais, which demonstrates its relevance to the community.

The target population of this study consisted of women aged 60 years or older who were referred to CRASI for care by the FSH teams of the municipalities in northern Minas Gerais and underwent BD diagnosis analysis. The initial proposal for the study would be restricted to the last six years. However, considering that during the Covid-19 pandemic, the service remained closed for a long time and had its activities reduced for a few subsequent months, the data collection period was extended to 10 years.

### Sample Size Calculation

To calculate the sample size, the proportion of BD tests performed in that service in the two years preceding data collection was considered. An estimated prevalence of 50% was assumed for the event studied (as this value provides the largest sample size), a confidence level of 95%, and a sampling error of 5%^(12)^.

### Data Collection

Data collection was performed using medical records, selected at random and respecting the previously defined inclusion criteria (older adult women who underwent the BD tests at the unit, referred by the FSH teams, regardless of the reason for referral). The form, developed by the researchers, included questions on sociodemographic aspects, Body Mass Index (BMI), lifestyle habits, health care, as well as morbidities reported and recorded in the medical records. The researchers categorized age into two ranges (60 to 79 years and 80 years or older) and education into up to four years and more than four years of completed studies. BMI was also assessed in two categories: less than 27.0 (underweight and normal weight) and ≥ 27.0 (overweight)^(13)^. Smoking, alcoholism, and regular/habitual coffee consumption were defined by self-report (recorded in medical records) and dichotomized into two categories (yes/no). Older adult women who reported not practicing physical activity on a regular basis, according to data from medical records, were considered sedentary. The history of morbidities was considered: systemic arterial hypertension (SAH), diabetes mellitus (DM), depression, dementia, previous fracture, osteoarthritis/arthritis, and thyroid diseases. Polypharmacy was recorded for patients regularly using five or more medications per day^(14)^.

Osteoporosis, a dependent variable, was diagnosed in the presence of a T-score ≤ -2.5 standard deviations^(15)^. Using a Discovery dual-energy densitometer, bone mineral density was measured, checking the values of the lumbar spine and femoral neck (Ward’s triangle). The tests, whose results were automatically analyzed and expressed in g/cm^2^, followed the same technical standard, having been conducted by the same professional and with the system controlled by computer.

### Data Analysis and Processing

After descriptive analysis, associations were verified using bivariate analyses based on Pearson’s chi-square test. At this stage, variables that were associated with osteoporosis up to a level of 20% (p ≤ 0.20) were selected for binary logistic regression analysis, and odds ratios and their respective 95% confidence intervals (95% CI) were obtained. For the final model, only variables with a significance level of up to 5% (p ≤ 0.05) were retained.

### Ethical Aspects

All ethical aspects were respected and the research was conducted in accordance with Resolution No. 466/2012 of the National Health Council. The use of medical records was authorized by the institution based on the Data Use Agreement (TCUD). The research project, registered under CAAE 45544521.6.0000. 5109, was approved by the Research Ethics Committee of the Pitágoras Integrated Faculties of Montes Claros, under opinion No. 4,679,222 of the Research Ethics Committee (CEP) and its amendment by opinion No. 5,839,913.

## RESULTS

A total of 647 women with the appropriate profile for the study objective were identified, who underwent BD examination between 2015 and 2024. The age of the group ranged from 63 to 101 years, with the predominant age group being 70 to 79 years (44.7%). Almost a quarter of the participating women reported being illiterate, and slightly more than 50% had between one and four years of schooling. In terms of BMI, more than 40% of the group was classified as overweight or obese, and a sedentary lifestyle was identified in more than 70% of the study participants (Table 1).

**Table 1:** Sociodemographic characteristics and lifestyle habits of older adult women assisted at a Reference Center for Older adult Health Care – Montes Claros, MG, Brazil, 2015–2024.

Variables	n	%
Age (years)		
60–69	120	18.5
70–79	289	44.7
≥ 80	238	36.8
Education		
Illiterate	152	23.5
1–4 years	352	54.4
5–8 years	108	16.7
≥ 9 years	25	3.9
No information	10	1.5
BMI		
≤ 22.0	115	17.8
22.1–26.9	219	33.8
≥ 27.0	285	44.0
No information	28	4.3
Smoking		
Past/current smoking	69	10.7
Non-smoker	578	89.3
Alcoholism		
Past/current alcoholism	64	9.9
Non-drinker	583	90.1
Sedentary lifestyle		
Yes	456	70.5
No	191	29.5
Excessive coffee consumption		
Yes	239	36.9
No	408	63.1

Source: Prepared by the authors

Regarding the morbidities recorded in medical records, the most common was hypertension (78.8%), followed by depression (51.6%). More than 40% of the women evaluated used five or more medications regularly per day (Table 2).

**Table 2:** Comorbidities of older adult women treated at a Center for Health Care for the Elderly – Montes Claros MG, Brazil, 2015–2024.

Variables	n	%
Systemic arterial hypertension	510	78.8
Diabetes mellitus	241	37.2
Dementia	248	38.3
Depression	334	51.6
Polypharmacy	280	43.3
Previous fracture	170	26.3
Arthritis/Osteoarthritis	302	46.7
Thyroid diseases	108	16.7

Source: Prepared by the authors.

The results of the BD tests revealed values compatible with osteoporosis for 300 women (46.4%). Osteopenia was identified in 239 older adult women (36.9%), and normal results were observed in only 108 (16.7%) of the women evaluated.

Table 3 presents the results of the bivariate analyses. Some data regarding the variables of education and BMI were not available at the time of collection and were considered omitted so as not to interfere with the analysis. The presence of multicollinearity among the continuous variables was evaluated using the correlation matrix, with no coefficients greater than 0.7 observed. For the final model, only variables with p < 0.05 were retained.

**Table 3:** Bivariate analysis of factors associated with osteoporosis in older adult women treated at a Center for Health Care for the Elderly – Montes Claros, MG, Brazil, 2015–2024.

Variable	Osteoporosis				p-value*
	Presence		Absence		
Education (years of study)					0.127
≤ 4	242	48.0	262	52.0	
> 4	54	40.6	79	59.4	
Age (years)					0.001
≥ 80	130	54.6	108	45.4	
< 80	170	41.6	239	58.4	
Body Mass Index					<0.001
< 27	182	54.3	153	45.7	
≥ 27	105	36.8	180	63.2	
Smoking					0.999
Past/Present	32	46.4	37	53.6	
Non-smoker	268	46.4	310	53.6	
Alcoholism					0.727
Past/Present	31	18.4	33	51.6	
Non-drinker	269	46.1	314	53.9	
Coffee consumption					0.134
Yes	120	50.2	119	49.8	
No	180	44.1	228	55.9	
Sedentarism					0.071
Yes	201	44.1	255	55.9	
No	99	51.8	92	48.2	
Polypharmacy					0.411
Yes	135	48.2	145	51.8	
No	165	45.0	202	55.0	
High Blood Pressure					0.776
Yes	235	46.1	275	53.9	
No	65	47.4	72	52.6	
Diabetes Mellitus					0.055
Yes	100	41.5	141	58.5	
No	200	49.3	206	50.7	
Depression					0.162
Yes	146	43.7	188	56.3	
No	154	49.2	159	50.8	
Dementia					0.870
Yes	116	46.8	132	53.2	
No	184	46.1	215	53.9	
Previous fracture					0.199
Yes	86	50.6	84	49.4	
No	214	44.9	263	55.1	
Arthritis/osteoarthritis					0.208
Yes	148	49.0	154	51.0	
No	152	44.1	193	55.9	
Thyroid disorders					0.407
Yes	54	50.0	54	50.0	
No	246	45.6	293	54.4	

Source: Prepared by the authors.

Based on these analyses, the variables education, age, BMI, coffee consumption, physical inactivity, diabetes mellitus, depression, and history of previous fractures were evaluated together using binary logistic regression. After multiple analysis, the variables that remained associated with osteoporosis were age equal to or greater than 80 years (p = 0.006; OR = 1.60; 95% CI: 1.14–2.24) and BMI < 27 (p < 0.001; OR = 1.97; 95% CI: 1.42–2.74). The Hosmer-Lemeshow test indicated a good fit of the model at all stages (p > 0.05), suggesting that the predictions do not differ significantly from the observed values.

## DISCUSSION

Osteopenia and osteoporosis were clinical conditions with high prevalence in the study population. These data are consistent with other studies that have demonstrated a high prevalence of osteoporosis in the female population. The literature reports a progressive increase in bone loss in women over 50 years of age, reaching two-thirds of the female population at 90 years of age^(16)^. Although this is not a population-based study, the similar results highlight the magnitude of the problem. Osteoporosis, a silent disease that particularly affects women after menopause, is the main cause of fractures in people over 50. In addition, it is associated with a significant increase in morbidity and mortality among patients with this clinical condition^(17)^.

In a study conducted at a specialized clinic in southern Brazil, researchers found that 49.8% of participants had osteopenia and 13.7% had osteoporosis. The results are similar to those found in the present study, and the differences observed may reflect the younger population evaluated^(9)^.

A study conducted in India with women aged 50 to 80 who underwent densitometry testing found that 37.5% had osteoporosis and 44.7% had osteopenia, indicating a high percentage of women with impaired bone calcification^(15)^.

It should be noted that the studies cited were conducted on women evaluated in densitometry clinics. Considering the general population, an American study reported that, in 2017–2018, the age-adjusted prevalence of osteoporosis among women aged 50 years or older was 19.6%, while the prevalence of osteopenia in the same age group was 51.5%. Compared to previous years, the authors observed an increase in the prevalence of the condition for the general population^(18)^. In the context of Latin America, the literature points out that osteoporosis is a global health condition that affects between 30% and 50% of people over 50 years of age^(19)^.

In the present study, after multiple analyses, only two variables were identified as independently associated with the development of bone demineralization. These were advanced age, especially in long-lived older adult women aged 80 years or older, and a body mass index (BMI) of less than 27 kg/m^2^. The other variables evaluated were not retained in the final model.

Regarding age, the existing literature indicates that aging is a risk factor for the development of osteoporosis. The result obtained in the sample studied is consistent with research that has shown a progressive loss of bone mass, especially among older adult women aged 80 years or older. Other epidemiological studies have also shown that the prevalence of osteoporosis-related complications increases significantly with advancing age, especially with regard to the occurrence of fragility fractures^(20,21)^.

A BMI below 27 kg/m^2^ has been associated with osteoporosis, and individuals with a BMI below this threshold are more likely to develop the disease compared to those with a BMI equal to or above this threshold. In a study conducted by Martini et al.^(20)^, which considered body mass index (BMI) as an isolated variable, it was observed that individuals with a BMI below 25 kg/m^2^ had an almost three times greater chance of suffering a fragility fracture. It is important to note that this cutoff point differs slightly from the values traditionally used to define overweight in adult patients. However, it is compatible with the normal limits expected for the older adult population, according to guidelines accepted by the Ministry of Health^(22,23)^.

It should be noted that excessive BMI is classically associated with several health risks, including the development of type 2 diabetes, non-alcoholic fatty liver disease, heart disease, and obstructive sleep apnea^(22,23)^. In addition, obesity can trigger a chronic inflammatory response, which can also increase the likelihood of osteoporosis and fragility fractures. Thus, an appropriate, but not excessive, BMI can allow older adults to have better bone mineral density, balancing the benefits for bone health with the prevention of other health problems associated with obesity^(22,24)^.

In addition to the factors mentioned as being associated with the presence of osteoporosis, it is important to note that some variables identified in other studies were not found to be associated with decreased bone mineral density in the older adult women evaluated. There were no results to support, for example, an association between certain lifestyle habits such as physical inactivity, smoking, and alcohol consumption and the occurrence of osteoporosis. It is possible that this lack of association is due to inadequate recording of such variables in medical records.

A study conducted in Picos (Piauí, Brazil), although it did not show a direct association between alcoholism and osteoporosis, showed that alcohol consumption was associated with a higher risk of fractures in the population studied. The study’s researchers suggest that excessive alcohol consumption may be linked to reduced bone mineral density and bone quality because of oxidative stress caused by the substance. In addition, acute alcohol intoxication has the potential to increase the risk of falls in this population^(25)^. It should be noted that the samples evaluated in both this study and the one conducted in Picos (PI) are composed exclusively or predominantly of women, among whom there is low reported alcohol consumption, which may have contributed to the results found.

An Italian study revealed a positive and equivalent association between active and passive smoking and susceptibility to osteoporosis^(26)^. In the current study, this relationship was not identified. However, it should be noted that, as with alcoholism, the proportion of women who reported tobacco use is considerably small.

The association between physical activity, assessed by recording walks lasting more than one hour, and the occurrence of osteoporosis was pointed out in a recent Chinese study^(27)^. This association was reiterated in another study that assessed the relationship between osteoporosis and healthy lifestyles in the older adult. Physical activity was highlighted positively, while alcohol abuse and smoking showed negative impacts on bone quality^(28)^.

The results did not establish an association between coffee consumption and reduced bone mineral density in the population evaluated. Although it is a variable recorded in some studies, there is uncertainty in the literature about the relationship between osteoporosis and coffee and tea consumption^(27)^. On this issue, a systematic review study concluded that there may be a dose-dependent relationship between coffee consumption, osteoporosis, and the incidence of hip fractures. The authors point out that the definition of high or low coffee consumption varies between studies and note that further research with dedicated designs is needed to confirm the independent effects of coffee consumption on bone health^(29)^.

The association between osteoporosis and polypharmacy, recorded in a study conducted in the north of the country^(30)^, was not observed in this study. This situation probably reflects some regional particularity, but it is possible that the way patients were allocated to the study also explains the observed divergence. It is possible that polypharmacy is an indicator of other conditions inherent to the aging process, with an increase in comorbidities, including osteoporosis.

Although conducting the study at a referral center for older adult health may represent a limitation in terms of generalizing the results, it should be noted that the choice of location provided access to medical records and test results collected and performed by qualified professionals, which allowed for greater accuracy and quality of the findings. In addition, the sample studied is representative, given that it consists of patients referred by primary care services, which was a criterion defined in advance for the inclusion of patients in the study. As this is a cross-sectional study, it is not possible to establish causal relationships between the variables analyzed. Furthermore, reliance on information recorded in medical records led to the loss of some data, which may represent an information bias, which was minimized through statistical analysis.

Although the analysis of medical records showed that most patients used bisphosphonates irregularly, especially alendronate, this finding was not investigated in the present study. Aspects of osteoporosis treatment, such as therapeutic adherence, patients’ understanding of the disease, and factors associated with inappropriate medication use, were not evaluated, which represents a limitation of this study and highlights a knowledge gap to be explored in future research.

## CONCLUSION

The study showed a high prevalence of osteopenia and osteoporosis in the population evaluated. It was observed that advanced age and low body mass index (BMI) represent significant risk factors for the development of osteoporosis in women. These findings suggest the need for more rigorous surveillance and preventive interventions targeting older women, especially those with low body weight. Thus, public policies related to the care of the older adult, as well as health professionals, should prioritize the implementation of health promotion and prevention actions, including regular physical activity and the adoption of a balanced diet to achieve an adequate BMI, thereby contributing to the reduction of the incidence of osteoporosis and its complications. In addition, more longitudinal studies are needed to gain a deeper understanding of the mechanisms related to these associations.

## Data Availability

The entire dataset supporting the results of this study is available upon request to the corresponding author.
