# Supplementary material for: Factors associated with osteoporosis in older adult women treated at a referral center
Source: Rev Esc Enferm USP. 2026 Jun 1;60:e20250489. doi: 10.1590/1980-220X-REEUSP-2025-0489en (PMC13232736; doi:10.1590/1980-220X-REEUSP-2025-0489en)
Supplement: Translation

# Translation: Fatores associados à osteoporose em mulheres idosas assistidas em um centro de referência

**DOI:** 10.1590/1980-220X-REEUSP-2025-0489pt

**Authors:** Maria Suzana Marques, Luiza Almeida Perdigão, Thomaz de Figueiredo Braga Colares, Ely Carlos Pereira de Jesus, Luciana Colares Maia, Antônio Prates Caldeira

**Affiliations:** 1 Universidade Estadual de Montes Claros, Montes Claros, MG, Brazil.

**Keywords:** Osteoporose, Idoso, Fatores de risco.

## Abstract

**Objetivo:**

Analisar os fatores associados à osteoporose em mulheres idosas assistidas em um centro de atenção à saúde do idoso, no norte de Minas Gerais.

**Método:**

Trata-se de um estudo transversal e analítico que envolveu 647 mulheres com 60 anos ou mais, submetidas à densitometria óssea entre os anos de 2015 e 2024. Os dados foram coletados a partir de prontuários médicos, incluindo informações sociodemográficas, clínicas e de hábitos de vida. A osteoporose foi diagnosticada por meio de T-score ≤ −2,5. Foram realizadas análises bivariadas e regressão logística binária.

**Resultados:**

Os resultados indicaram uma prevalência de osteoporose de 46,4% entre as participantes. A análise multivariada revelou que idade igual ou superior a 80 anos (OR = 1,60; IC 95%:1,14–2,24) e índice de massa corporal (IMC) inferior a 27 kg/m^2^ (OR = 1,97; IC 95%:1,42–2,74) são fatores associados à osteoporose.

**Conclusão:**

Esses achados demonstram a elevada prevalência de osteoporose na população estudada, bem como a necessidade de implementação de estratégias e intervenções preventivas, especialmente em idosas com baixo peso corporal.

## INTRODUÇÃO

A osteoporose é a principal doença osteometabólica em pessoa idosa. É caracterizada pela redução da densidade mineral óssea, com importante comprometimento da microarquitetura dos ossos, o que leva a um aumento do risco de quedas e fraturas^(1,2)^. Trata-se de uma condição de alta prevalência mundial, especialmente em países europeus e africanos. Nesse sentido, estudo de metanálise evidencia que a prevalência global de osteoporose é de 18,3% (IC 95% 16,2–20,7), sendo mais elevada entre mulheres, alcançando taxas de 23,1% (IC 95% 19,8–26,9)^(3)^.

O acelerado processo de envelhecimento da população brasileira^(4)^ aponta para um crescente número de mulheres idosas com demanda por serviços de saúde, o que demonstra a relevância da osteoporose enquanto um iminente problema de saúde pública. Destaca-se que a osteoporose não possui manifestações clínicas evidentes até que a pessoa apresente uma fratura. Para as pessoas idosas, as fraturas estão associadas ao aumento da morbimortalidade e levam a um importante comprometimento da qualidade de vida, tanto para os próprios pacientes, como para suas famílias, que passam a lidar com maiores limitações^(2)^. Esse fato ressalta a importância do diagnóstico precoce da doença, a partir de avaliação oportuna da densidade mineral óssea, além do tratamento precoce, antes que as fraturas ocorram^(5)^. Estudos nacionais, embora pouco frequentes, destacam a relevância do tema^(6,7)^.

Um aspecto limitante para a pesquisa sobre a osteoporose diz respeito à alocação de pacientes. Tendo em vista que estudos de base populacional são difíceis de serem conduzidos e implicam maiores custos. Nesse sentido, alguns estudos abordam a prevalência e os fatores associados a partir da população submetida ao exame de densitometria óssea (DO), considerado o padrão-ouro para o diagnóstico da doença^(8,9)^. Nesse contexto, os centros de atenção à saúde do idoso desempenham um papel importante na identificação oportuna e na gestão da osteoporose. Em toda a região norte do estado de Minas Gerais, um único centro de atenção à saúde do idoso é responsável pela realização de exames de DO, via Sistema Único de Saúde (SUS). Esse centro atende a macrorregião do norte de Minas, que abrange 86 municípios e população que ultrapassa 255.000 pessoas idosas^(10,11)^.

Considerando que, nos últimos anos, esforços têm sido feitos para ampliar o conhecimento sobre a epidemiologia da osteoporose no Brasil, de maneira a melhorar a eficácia da prevenção da doença, e que ainda existem poucas pesquisas sobre o tema na macrorregião de Montes Claros, Minas Gerais^(12)^, este estudo objetivou analisar os fatores associados à osteoporose em mulheres idosas assistidas em um centro de atenção à saúde do idoso no norte de Minas Gerais.

## MÉTODO

### Desenho do Estudo

Trata-se de um estudo transversal e analítico.

### Local, População e Critérios de Seleção

Foi avaliada a população de mulheres idosas encaminhadas pelas equipes de Estratégia Saúde da Família (ESF) ao Centro de Referência em Assistência à Saúde dos Idosos (CRASI) para a realização de exames de DO. Este centro está vinculado ao Hospital Universitário Clemente de Faria e realiza atendimentos em saúde exclusivamente pelo SUS. O serviço é constituído por uma equipe de profissionais de diferentes áreas e formações, prestando assistência em saúde de caráter interprofissional. O CRASI atende aproximadamente 1.000 pessoas por mês e realiza cerca de 400 exames de DO, abrangendo pacientes referenciados pelas equipes de Atenção Primária dos municípios do norte de Minas Gerais, o que demonstra sua relevância para a comunidade.

Logo, a população-alvo deste estudo consistiu em mulheres com idade igual ou superior a 60 anos, referenciadas para atendimento no CRASI pelas equipes de ESF dos municípios do norte de Minas Gerais e submetidas ao exame de DO. A proposta inicial do estudo seria restrita aos últimos seis anos. Todavia, considerando que, durante a pandemia de Covid-19, o serviço permaneceu fechado por longo tempo e teve suas atividades reduzidas por alguns meses subsequentes, o período de coleta de dados foi ampliado para 10 anos.

### Cálculo do Tamanho da Amostra

Para o cálculo do tamanho da amostra, considerou-se a proporção de exames de DO realizados no referido serviço pelo tempo de dois anos que precederam a coleta de dados. Assumiu-se uma prevalência estimada de 50% para o evento estudado (por ser um valor que fornece o maior número amostral), um nível de confiança de 95% e um erro amostral de 5%^(12)^.

### Coleta de Dados

A coleta de dados foi feita em prontuários médicos, selecionados aleatoriamente e respeitando os critérios de inclusão previamente definidos (idosas, que realizaram o exame na unidade, que foram referenciadas pelas equipes de ESF, independente do motivo de encaminhamento). O formulário, elaborado pelos pesquisadores, incluiu questões sobre aspectos sociodemográficos, Índice de Massa Corporal (IMC), hábitos de vida, cuidados de saúde, além de morbidades referidas e registradas nos prontuários. Os pesquisadores categorizaram a idade em duas faixas: 60 a 79 anos e 80 anos ou mais; já a escolaridade: até quatro anos e mais de quatro anos de estudos completos. O IMC foi avaliado também em duas categorias: inferior a 27,0 (baixo peso e peso normal) e ≥ 27,0 (excesso de peso)^(13)^. O tabagismo, etilismo e consumo regular/habitual de café foram definidos pelo autorrelato (registrados em prontuários) e dicotomizados em duas categorias (sim/não). Além do mais, considerou-se sedentária a idosa que relatou não praticar atividade física de forma regular, conforme os dados dos prontuários. Tal qual, ainda foi considerado o histórico das morbidades: hipertensão arterial sistêmica (HAS), diabetes mellitus (DM), depressão, demência, fratura prévia, artrose/artrite e doenças da tireoide. Por fim, o registro de polifarmácia foi feito para pacientes em uso regular de cinco ou mais medicações por dia^(14)^.

A osteoporose, variável dependente, foi diagnosticada na presença de um T-score ≤ -2,5 desvios-padrão^(15)^. Utilizando-se um densitômetro de dupla energia *Discovery*, foi realizada a medição da densidade mineral óssea, verificando os valores da coluna lombar e do colo femoral (triângulo de Ward). Os exames, cujos resultados foram analisados automaticamente e expressos em g/cm^2^, seguiram o mesmo padrão técnico, tendo sido conduzidos por um mesmo profissional e com o sistema controlado por computador.

### Análise e Tratamento dos Dados

Após a análise descritiva, foram verificadas associações a partir de análises bivariadas e por intermédio do teste qui-quadrado de Pearson. Nessa etapa, as variáveis que se mostraram associadas à osteoporose até o nível de 20% (p ≤ 0,20) foram selecionadas para análise de regressão logística binária, sendo obtidas as Odds Ratios e seus respectivos Intervalos de Confiança de 95% (IC 95%). Para o modelo final, foram mantidas apenas as variáveis com nível de significância de até 5% (p ≤ 0,05).

### Aspectos Éticos

Todos os aspectos éticos foram respeitados e a pesquisa foi conduzida de acordo com a Resolução nº 466/2012 do Conselho Nacional de Saúde. A utilização dos prontuários foi autorizada pela instituição a partir do Termo de Compromisso de Utilização de Dados (TCUD). O projeto de pesquisa, registrado sob o CAAE 45544521.6.0000.5109, foi aprovado pelo Comitê de Ética em Pesquisa das Faculdades Integradas Pitágoras de Montes Claros, sob o parecer nº 4.679.222 do Comitê de Ética em Pesquisa (CEP) e a sua emenda foi aprovada pelo parecer n.º 5.839.913.

## RESULTADOS

Foram identificadas 647 mulheres com o perfil adequado ao objetivo do estudo, além de terem realizado o exame de DO entre os anos de 2015 e 2024. A idade do grupo variou de 63 a 101 anos, portanto a faixa etária predominante foi de 70 a 79 anos (44,7%). Quase um quarto das mulheres participantes se referiu como analfabeta e um pouco mais de 50% tinham escolaridade que variou de um a quatro anos de estudo. Em relação ao IMC, mais de 40% do grupo foi classificado como sobrepeso ou obesidade e o sedentarismo foi identificado em mais de 70% das participantes da pesquisa (Tabela 1).

**Tabela 1:** Características sociodemográficas e de hábitos de vida de idosas assistidas em um Centro de Referência em Assistência à Saúde dos Idosos – Montes Claros, MG, Brasil, 2015–2024.

Variáveis	n	%
Idade (anos)		
60–69	120	18,5
70–79	289	44,7
≥ 80	238	36,8
Escolaridade		
Analfabeto	152	23,5
1–4 anos	352	54,4
5–8 anos	108	16,7
≥ 9 anos	25	3,9
Sem informações	10	1,5
IMC		
≤ 22,0	115	17,8
22,1- 26,9	219	33,8
≥ 27,0	285	44,0
Sem informações	28	4,3
Tabagismo		
Tabagismo pregresso/atual	69	10,7
Não tabagista	578	89,3
Etilismo		
Etilismo pregresso/atual	64	9,9
Não etilista	583	90,1
Sedentarismo		
Sim	456	70,5
Não	191	29,5
Consumo excessivo de café		
Sim	239	36,9
Não	408	63,1

Fonte: Elaborada pelos autores.

Em relação às morbidades registradas em prontuários, a mais comum foi a HAS (78,8%), seguida pela depressão (51,6%). Mais de 40% das mulheres avaliadas faziam uso de cinco ou mais medicamentos de forma regular por dia (Tabela 2).

**Tabela 2:** Comorbidades de idosas assistidas em um Centro de Referência em Assistência à Saúde dos Idosos – Montes Claros, MG, Brasil, 2015–2024.

Variáveis	n	%
Hipertensão arterial sistêmica	510	78,8
Diabetes Mellitus	241	37,2
Demência	248	38,3
Depressão	334	51,6
Polifarmácia	280	43,3
Fratura prévia	170	26,3
Artrite/Artrose	302	46,7
Doenças da tireoide	108	16,7

Fonte: Elaborada pelos autores.

Os resultados dos exames de DO revelaram valores compatíveis com osteoporose para 300 mulheres (46,4%). Já a osteopenia foi identificada em 239 idosas (36,9%) e o resultado normal foi observado em apenas 108 (16,7%) das mulheres avaliadas.

A Tabela 3 apresenta os resultados das análises bivariadas. Alguns dados referentes às variáveis escolaridade e IMC não estavam disponíveis no momento da coleta e foram considerados como omissos para não interferirem na análise. Foi avaliada a presença de multicolinearidade, entre as variáveis contínuas, por meio da matriz de correlação, não sendo observados coeficientes superiores a 0,7. Para o modelo final, foram mantidas apenas as variáveis com p < 0,05.

**Tabela 3:** Análise bivariada dos fatores associados à osteoporose de idosas assistidas em um Centro de Referência em Assistência à Saúde dos Idosos – Montes Claros, MG, Brasil, 2015–2024.

Variável	Osteoporose				p-valor*
	Presente		Ausente		
Escolaridade (anos de estudo)					0,127
≤ 4	242	48,0	262	52,0	
> 4	54	40,6	79	59,4	
Idade (anos)					0,001
≥ 80	130	54,6	108	45,4	
< 80	170	41,6	239	58,4	
Índice de Massa Corporal					<0,001
< 27	182	54,3	153	45,7	
≥ 27	105	36,8	180	63,2	
Tabagismo					0,999
Pregresso/Atual	32	46,4	37	53,6	
Não tabagista	268	46,4	310	53,6	
Etilismo					0,727
Pregresso/Atual	31	18,4	33	51,6	
Não etilista	269	46,1	314	53,9	
Consumo de café					0,134
Sim	120	50,2	119	49,8	
Não	180	44,1	228	55,9	
Sedentarismo					0,071
Sim	201	44,1	255	55,9	
Não	99	51,8	92	48,2	
Polifarmácia					0,411
Sim	135	48,2	145	51,8	
Não	165	45,0	202	55,0	
Hipertensão Arterial					0,776
Sim	235	46,1	275	53,9	
Não	65	47,4	72	52,6	
Diabetes Mellitus					0,055
Sim	100	41,5	141	58,5	
Não	200	49,3	206	50,7	
Depressão					0,162
Sim	146	43,7	188	56,3	
Não	154	49,2	159	50,8	
Demência					0,870
Sim	116	46,8	132	53,2	
Não	184	46,1	215	53,9	
Fratura prévia					0,199
Sim	86	50,6	84	49,4	
Não	214	44,9	263	55,1	
Artrite/artrose					0,208
Sim	148	49,0	154	51,0	
Não	152	44,1	193	55,9	
Doenças da tireoide					0,407
Sim	54	50,0	54	50,0	
Não	246	45,6	293	54,4	

Fonte: Elaborada pelos autores.

A partir dessas análises, as variáveis: escolaridade, idade, IMC, consumo de café, sedentarismo, diabetes mellitus, depressão e registro de fratura prévia foram avaliadas conjuntamente por meio de regressão logística binária. Após análise múltipla, as variáveis que permaneceram associadas à osteoporose foram: idade igual ou superior a 80 anos (p = 0,006; OR = 1,60; IC 95%: 1,14–2,24) e IMC < 27 (p < 0,001; OR = 1,97; IC 95%: 1,42–2,74). O teste de Hosmer-Lemeshow indicou um bom ajuste do modelo em todas as etapas (p > 0,05), sugerindo que as previsões não diferem significativamente dos valores observados.

## DISCUSSÃO

A osteopenia e a osteoporose representaram condições clínicas com elevada prevalência na população estudada. Esses dados são compatíveis com outros estudos que demonstraram elevada prevalência de osteoporose na população feminina. Além do mais, a literatura registra um progressivo aumento de perda óssea em mulheres acima de 50 anos, chegando a atingir dois terços da população feminina aos 90 anos^(16)^. Embora este não seja um estudo de base populacional, os resultados similares destacam a magnitude do problema. A osteoporose, uma doença silenciosa e que afeta especialmente mulheres após a menopausa, é a principal responsável por fraturas em pessoas com mais de 50 anos. Além disso, está associada a um aumento significativo de morbimortalidade entre os pacientes com tal condição clínica^(17)^.

Em um estudo realizado em uma clínica especializada no sul do Brasil, os pesquisadores descobriram que 49,8% das participantes apresentavam osteopenia e 13,7% tinham osteoporose. Logo, os resultados são semelhantes aos encontrados no presente estudo e as diferenças observadas podem ser reflexo da população mais jovem avaliada^(9)^.

Ademais, uma pesquisa realizada na Índia, com mulheres de 50 a 80 anos que foram submetidas ao exame de densitometria, registrou que 37,5% apresentavam osteoporose e 44,7% apresentavam osteopenia, indicando um elevado percentual de mulheres com comprometimento da calcificação óssea^(15)^.

É preciso destacar que os estudos citados foram realizados a partir de mulheres avaliadas em clínicas de densitometria. Considerando a população em geral, um estudo americano registrou que, em 2017–2018, a prevalência ajustada por idade de osteoporose entre mulheres com 50 anos ou mais foi de 19,6%, enquanto a prevalência de osteopenia na mesma faixa etária foi de 51,5%. Comparativamente aos anos anteriores, os autores observaram o aumento da prevalência da condição para a população em geral^(18)^. No contexto da América Latina, a literatura aponta que a osteoporose é uma condição de saúde global que impacta entre 30% e 50% das pessoas com mais de 50 anos^(19)^.

No presente estudo, após análise múltipla, foram identificadas apenas duas variáveis associadas de maneira independente com o desenvolvimento da desmineralização óssea, sendo elas a idade avançada, especialmente quando se trata de idosas longevas (com 80 anos ou mais), e o índice de massa corporal (IMC) inferior a 27 kg/m^2^. As demais variáveis avaliadas não se mantiveram no modelo final.

De modo análogo, em relação à idade, a literatura existente aponta que o envelhecimento é um fator de risco para o desenvolvimento de osteoporose. O resultado obtido na amostra estudada é consistente com pesquisas que evidenciaram uma perda progressiva de massa óssea, principalmente entre idosas com 80 anos ou mais. Outros estudos epidemiológicos também demonstraram que a prevalência de complicações relacionadas à osteoporose aumenta significativamente com o avanço da idade, especialmente no que diz respeito à ocorrência de fraturas por fragilidade^(20,21)^.

O IMC inferior a 27 kg/m^2^ tem sido associado à osteoporose, sendo que indivíduos com IMC abaixo deste limiar têm maior probabilidade de apresentar a doença em comparação com aqueles com IMC igual ou superior. Em um estudo conduzido por Martini *et al*, que considerou o índice de massa corporal (IMC) como variável isolada, foi observado que indivíduos com IMC abaixo de 25 kg/m^2^ tiveram uma chance quase três vezes maior de sofrer fratura por fragilidade^(20)^. Por conseguinte, é importante notar que este ponto de corte difere ligeiramente dos valores tradicionalmente utilizados para definir sobrepeso em pacientes adultos. No entanto, ele é compatível com os limites de normalidade esperados para a população idosa, conforme diretrizes aceitas pelo Ministério da Saúde^(22,23)^.

Ressalta-se que o IMC excessivo é classicamente associado a diversos riscos à saúde, incluindo o desenvolvimento de diabetes tipo 2, doença hepática gordurosa não alcoólica, doenças cardíacas e apneia obstrutiva do sono^(22,23)^. Além disso, a obesidade pode desencadear uma resposta inflamatória crônica, o que pode também aumentar a possibilidade de osteoporose e fraturas por fragilidade. Dessa forma, um IMC apropriado, mas não excessivo, pode permitir que os idosos tenham uma melhor densidade mineral óssea, equilibrando os benefícios para a saúde óssea com a prevenção de outros problemas de saúde associados à obesidade^(22,24)^.

Além dos fatores mencionados como associados à presença de osteoporose, é relevante destacar que algumas variáveis apontadas em outros estudos não se mostraram associadas à diminuição da densidade mineral óssea das idosas avaliadas. Não houve resultados que sustentassem, por exemplo, a associação entre alguns hábitos de vida como sedentarismo, tabagismo e etilismo com a ocorrência de osteoporose. Entretanto, vale ressaltar que é possível que essa falta de associação seja decorrente do registro inadequado em prontuários em relação a tais variáveis.

Um estudo realizado em Picos (Piauí, Brasil), apesar de não ter apresentado associação direta entre etilismo e osteoporose, mostrou que a ingestão de bebidas alcoólicas foi associada a um maior risco de fraturas na população estudada. Os pesquisadores do estudo sugerem que o consumo excessivo de álcool pode estar ligado à redução da densidade mineral óssea e da qualidade do osso, como resultado do estresse oxidativo causado pela substância. Além disso, a intoxicação aguda pelo álcool tem o potencial de aumentar o risco de quedas nesta população^(25)^. Há de se ressaltar que as amostras avaliadas, tanto neste estudo como o realizado em Picos (PI), são formadas exclusivamente ou predominantemente por mulheres, dentre as quais há baixo relato de consumo de álcool, o que pode ter contribuído para os resultados encontrados.

Ainda, uma pesquisa italiana revelou associação positiva e equivalente entre fumo ativo e passivo na suscetibilidade à ocorrência de osteoporose^(26)^. No atual estudo, essa relação não foi identificada. Porém, salienta-se que da mesma maneira que o etilismo, a proporção de mulheres que relataram consumo de tabaco é consideravelmente pequena.

Já a associação entre a prática de atividade física, avaliada pelo registro de caminhadas com tempo superior a uma hora, e a ocorrência de osteoporose foi apontada em recente estudo chinês^(27)^. Essa associação foi reiterada em outro estudo que avaliou a relação entre osteoporose e estilos de vida saudáveis em idosos. Houve destaque positivo para a atividade física, enquanto abuso o de álcool e o tabagismo mostraram impactos negativos sobre a qualidade do osso^(28)^.

Os resultados não estabeleceram associação entre o consumo de café e a redução da densidade mineral óssea na população avaliada. Embora seja uma variável registrada em alguns estudos, existe uma indefinição na literatura sobre a relação entre a osteoporose e o consumo de café e chá^(27)^. Sobre essa questão, um estudo de revisão sistemática concluiu que pode existir uma relação dose-dependente entre o consumo de café, a osteoporose e a incidência de fratura de quadril. Os autores destacam que a definição de alto ou baixo consumo de café é variável entre os estudos e registram que novas pesquisas com desenhos dedicados são necessárias para confirmar os efeitos independentes do consumo de café na saúde óssea^(29)^.

A associação entre osteoporose e polifarmácia, registrada em pesquisa realizada no Norte do país^(30)^, não foi observada neste estudo. Tal situação retrata, provavelmente, alguma particularidade regional, mas é possível que a forma de alocação dos pacientes para a pesquisa também justifique a divergência observada. Há possibilidade de que a polifarmácia seja um indicador de outras condições inerentes ao processo de envelhecimento, com aumento de comorbidades, incluindo a osteoporose.

Embora a condução do estudo em um centro de referência em saúde do idoso possa representar uma limitação quanto à generalização dos resultados, há que se ressaltar que a escolha do local ofereceu a oportunidade de acesso aos dados de prontuários e aos resultados de exames coletados e executados por profissionais qualificados, o que permitiu maior precisão e qualidade dos achados. Além disso, a amostra estudada é representativa, tendo em vista que se trata de pacientes referenciadas pelos serviços de Atenção Primária, o que foi um critério definido previamente para a inclusão das pacientes no estudo. Por se tratar de uma pesquisa transversal, não é possível estabelecer relações de causalidade entre as variáveis analisadas. Além disso, a dependência das informações registradas em prontuários ocasionou a perda de alguns dados, o que pode representar um viés de informação, minimizado por meio da análise estatística.

Embora a análise dos prontuários tenha mostrado que a maioria das pacientes fazia uso irregular de bifosfonatos, especialmente alendronato, esse achado não foi objeto de investigação no presente estudo. Aspectos do tratamento da osteoporose não foram avaliados, como: adesão terapêutica, compreensão dos pacientes sobre a doença e fatores associados ao uso inadequado da medicação, o que representa uma limitação deste estudo e evidencia uma lacuna de conhecimento a ser explorada em pesquisas futuras.

## CONCLUSÃO

Por fim, a pesquisa mostrou elevada prevalência de osteopenia e osteoporose na população avaliada. Observou-se, ainda, que a idade avançada e o baixo índice de massa corporal (IMC) representam fatores de risco significativos para o desenvolvimento de osteoporose em mulheres. Esses achados sugerem a necessidade de vigilância mais rigorosa e intervenções preventivas direcionadas a idosas, em especial àquelas com baixo peso corporal. Assim, as políticas públicas relacionadas ao cuidado da pessoa idosa, bem como os profissionais de saúde devem atuar prioritariamente na implementação de ações de promoção e prevenção em saúde, incluindo a prática regular de atividade física e a adoção de dieta balanceada para alcançar IMC adequado e, por conseguinte, contribuir para a redução da incidência de osteoporose e suas complicações. Além disso, são necessários mais estudos longitudinais para compreender de maneira mais profunda os mecanismos relacionados a essas associações.

## Data Availability

Todo o conjunto de dados que dá suporte aos resultados deste estudo está disponível mediante solicitação ao autor correspondente.

# Translation: Factores asociados a la osteoporosis en mujeres mayores tratadas en un centro de referencia

**DOI:** 10.1590/1980-220X-REEUSP-2025-0489es

**Authors:** Maria Suzana Marques, Luiza Almeida Perdigão, Thomaz de Figueiredo Braga Colares, Ely Carlos Pereira de Jesus, Luciana Colares Maia, Antônio Prates Caldeira

**Affiliations:** 1 Universidade Estadual de Montes Claros, Montes Claros, MG, Brazil.

**Keywords:** Osteoporosis, Anciano, Factores de Riesgo.

## Abstract

**Objetivo:**

Analizar los factores asociados a la osteoporosis en mujeres mayores atendidas en un centro de atención especializado, en el norte de Minas Gerais.

**Método:**

Se trata de un estudio transversal y analítico en el que participaron 647 mujeres a partir de 60 años, sometidas a densitometría ósea, entre los años 2015 y 2024. Los datos se recopilaron de sus historias clínicas, información sociodemográfica, clínica y hábitos de vida. La osteoporosis se diagnosticó mediante un T-score ≤ −2,5. Se realizaron análisis bivariados y regresión logística binaria.

**Resultados:**

Los resultados indicaron una prevalencia de osteoporosis del 46,4 % entre las participantes. El análisis multivariado reveló que la edad igual o superior a 80 años (OR = 1,60; IC del 95 %: 1,14–2,24) y el índice de masa corporal (IMC) inferior a 27 kg/m^2^ (OR = 1,97; IC del 95 %: 1,42–2,74) son factores que están asociados a la osteoporosis.

**Conclusión:**

Estos descubrimientos señalan la elevada prevalencia de la osteoporosis en la población estudiada, así como la necesidad de implementar estrategias e intervenciones preventivas, especialmente en mujeres mayores con bajo peso corporal.

## INTRODUCCIÓN

La osteoporosis es la principal enfermedad osteometabólica en las personas de edad avanzada. Se caracteriza por una reducción de la densidad mineral ósea, con un importante deterioro de la microarquitectura de los huesos, lo que conlleva un aumento del riesgo de caídas y fracturas^(1,2)^. Se trata de una afección de alta prevalencia mundial, especialmente en países europeos y africanos. En este sentido, un metaanálisis evidencia que la prevalencia global de la osteoporosis es del 18,3 % (IC 95 %: 16,2–20,7), siendo más elevada entre las mujeres, con tasas que alcanzan el 23,1 % (IC 95 %: 19,8–26,9)^(3)^.

El acelerado proceso de envejecimiento de la población brasileña^(4)^ apunta a un número creciente de mujeres de edad avanzada con demanda de servicios de salud, lo que demuestra la relevancia de la osteoporosis como un inminente problema de salud pública. Cabe destacar que la osteoporosis no presenta manifestaciones clínicas evidentes hasta que la persona sufre una fractura. En las personas de edad avanzada, las fracturas se asocian con un aumento de la morbilidad y la mortalidad y conllevan un importante deterioro de la calidad de vida, tanto para los propios pacientes como para sus familias, que deben lidiar con mayores limitaciones^(2)^. Este hecho resalta la importancia del diagnóstico precoz de la enfermedad, a partir de una evaluación oportuna de la densidad mineral ósea, además del tratamiento precoz, antes de que se produzcan las fracturas*(5)*. Los estudios nacionales, aunque poco frecuentes, destacan la relevancia del tema^(6,7)^.

Un aspecto limitante para la investigación sobre la osteoporosis se refiere a la selección de pacientes. Teniendo en cuenta que los estudios poblacionales son difíciles de llevar a cabo e implican mayores costos. En este sentido, algunos estudios abordan la prevalencia y los factores asociados a partir de la población sometida a la densitometría ósea (DO), considerada el estándar de referencia para el diagnóstico de la enfermedad^(8,9)^. En este contexto, los centros de atención de salud para personas mayores desempeñan un papel importante en la identificación oportuna y el manejo de la osteoporosis. En toda la región norte del estado de Minas Gerais, un único centro de atención de salud para personas mayores se encarga de realizar los exámenes de DO, a través del Sistema Único de Salud (SUS). Este centro atiende a la macrorregión del norte de Minas, que abarca 86 municipios y una población que supera las 255 000 personas mayores^(10,11)^.

Teniendo en cuenta que, en los últimos años, se han realizado esfuerzos para ampliar el conocimiento sobre la epidemiología de la osteoporosis en Brasil, con el fin de mejorar la eficacia de la prevención de la enfermedad, y que aún existen pocas investigaciones sobre el tema en la macrorregión de Montes Claros, Minas Gerais^(12)^, este estudio tuvo como objetivo analizar los factores asociados a la osteoporosis en mujeres de edad avanzada atendidas en un centro de atención de salud para personas mayores en el norte de Minas Gerais.

## MÉTODO

### Diseño del Estudio

Se trata de un estudio transversal y analítico.

### Lugar, Población y Criterios de Selección

Se evaluó a la población de mujeres de edad avanzada derivadas por los equipos de la Estrategia de Salud de la Familia (ESF) al Centro de Referencia en Atención de Salud para Personas de Edad (CRASI en portugués) para la realización de exámenes de osteoporosis. Este centro está vinculado al Hospital Universitario Clemente de Faria y brinda atención de salud exclusivamente a través del SUS. El servicio está integrado por un equipo de profesionales de diferentes áreas y formaciones, que prestan asistencia sanitaria de carácter interprofesional. El CRASI atiende a aproximadamente 1.000 personas al mes y realiza cerca de 400 exámenes de DO, abarcando a pacientes derivados por los equipos de Atención Primaria de los municipios del norte de Minas Gerais, lo que demuestra relevancia para la comunidad.

Por lo tanto, la población objetivo de este estudio consistió en mujeres de 60 años o más, derivadas para recibir atención en el CRASI por los equipos de ESF de los municipios del norte de Minas Gerais y sometidas al examen de DO. La propuesta inicial del estudio se limitaría a los últimos seis años. Sin embargo, considerando que, durante la pandemia de Covid-19, el servicio permaneció cerrado durante un largo tiempo y sus actividades se redujeron durante algunos meses posteriores, el período de recolección de datos se amplió a 10 años.

### Cálculo del Tamaño de la Muestra

Para el cálculo del tamaño de la muestra, se consideró la proporción de exámenes de DO realizados en dicho servicio durante los dos años que precedieron a la recolección de datos. Se asumió una prevalencia estimada del 50 % para el evento estudiado (por ser un valor que proporciona el mayor tamaño de la muestra), un nivel de confianza del 95 % y un error muestral del 5 %^(12)^.

### Recolección de Datos

La recolección de datos se realizó en historias clínicas, seleccionadas al azar y respetando los criterios de inclusión previamente definidos (mujeres de edad avanzada, que se realizaron el examen en la unidad, que fueron derivadas por los equipos de ESF, independientemente del motivo de la derivación). El formulario, elaborado por los investigadores, incluía preguntas sobre aspectos sociodemográficos, índice de masa corporal (IMC), hábitos de vida, cuidados de salud, además de morbilidades referidas y registradas en los expedientes médicos. Los investigadores clasificaron la edad en dos grupos: de 60 a 79 años y 80 años o más; en cuanto a la escolaridad: hasta cuatro años y más de cuatro años de estudios completos. El IMC también se evaluó en dos categorías: inferior a 27,0 (bajo peso y peso normal) y superior a 27,0 (sobrepeso)^(13)^. El tabaquismo, el alcoholismo y el consumo regular/habitual de café se definieron mediante autoinforme (registrado en los expedientes médicos) y se dicotomizaron en dos categorías (sí/no). Además, se consideró sedentaria a la persona mayor que informó no practicar actividad física de manera regular, según los datos de los expedientes médicos. Asimismo, se consideró el historial de morbilidades: hipertensión arterial sistémica (HAS), diabetes mellitus (DM), depresión, demencia, fractura previa, artrosis/artritis y enfermedades tiroideas. Por último, se registró la polifarmacia en pacientes que tomaban regularmente cinco o más medicamentos al día^(14)^.

La osteoporosis, variable dependiente, se diagnosticó ante la presencia de un T-score ≤ -2,5^(15)^. Utilizando un densitómetro de doble energía *Discovery*, se realizó la medición de la densidad mineral ósea, verificando los valores de la columna lumbar y del cuello femoral (triángulo de Ward). Los exámenes, cuyos resultados se analizaron automáticamente y se expresaron en g/cm^2^, siguieron el mismo protocolo técnico, habiendo sido realizados por un mismo profesional y con el sistema controlado por computadora.

### Análisis y Tratamiento de los Datos

Tras el análisis descriptivo, se verificaron las asociaciones mediante análisis bivariados y a través de la prueba de chi-cuadrado de Pearson. En esta etapa, las variables que mostraron una asociación con la osteoporosis hasta un nivel del 20 % (p ≤ 0,20) fueron seleccionadas para el análisis de regresión logística binaria, obteniéndose las razones de probabilidades (Odds Ratios) y sus respectivos intervalos de confianza del 95 % (IC 95 %). Para el modelo final, se mantuvieron únicamente las variables con un nivel de significación de hasta el 5 % (p ≤ 0,05).

### Aspectos Éticos

Se respetaron todos los aspectos éticos y la investigación se llevó a cabo de conformidad con la Resolución n.º 466/2012 del Consejo Nacional de Salud. El uso de los expedientes médicos fue autorizado por la institución mediante el Término de Compromiso de Utilización de Datos (TCUD). El proyecto de investigación, registrado bajo el CAAE 45544521.6.0000. 5109, fue aprobado por el Comité de Ética en Investigación de las Facultades Integradas Pitágoras de Montes Claros, bajo el dictamen n.º 4.679.222 del Comité de Ética en Investigación (CEP) y su enmienda fue aprobada por el dictamen n.º 5.839.913.

## RESULTADOS

Se identificaron 647 mujeres con el perfil adecuado para el objetivo del estudio, además de haberse realizado el examen de DO entre los años 2015 y 2024. La edad del grupo varió de 63 a 101 años, por lo que el rango de edad predominante fue de 70 a 79 años (44,7 %). Casi una cuarta parte de las mujeres participantes se identificó como analfabeta y poco más del 50 % tenía un nivel de escolaridad que oscilaba entre uno y cuatro años de estudios. En cuanto al IMC, más del 40 % del grupo fue clasificado como con sobrepeso u obesidad y se identificó sedentarismo en más del 70 % de las participantes en la investigación (Tabla 1).

**Tabla 1:** Características sociodemográficas y de hábitos de vida de mujeres mayores atendidas en un Centro de Referencia en Asistencia Sanitaria para Personas Mayores – Montes Claros, MG, Brasil, 2015–2024.

Variables	n	%
Edad (años)		
60–69	120	18,5
70–79	289	44,7
≥ 80	238	36,8
Nivel educativo		
Analfabeto	152	23,5
1 – 4 años	352	54,4
5 – 8 años	108	16,7
≥ 9 años	25	3,9
Sin datos	10	1,5
IMC		
≤ 22,0	115	17,8
22,1-26,9	219	33,8
≥ 27,0	285	44,0
Sin datos	28	4,3
Tabaquismo		
Tabaquismo pasado/actual	69	10,7
No fumador	578	89,3
Alcoholismo		
Alcoholismo pasado/actual	64	9,9
No bebedor	583	90,1
Sedentarismo		
Sí	456	70,5
No	191	29,5
Consumo excesivo de café		
Sí	239	36,9
No	408	63,1

Fuente: Elaborado por los autores.

En cuanto a las morbilidades registradas en las historias clínicas, la más común fue la hipertensión arterial (78,8 %), seguida de la depresión (51,6 %). Más del 40 % de las mujeres evaluadas tomaban cinco o más medicamentos de forma regular al día (Tabla 2).

**Tabla 2:** Comorbilidades de las mujeres mayores atendidas en un Centro de Referencia en Atención Sanitaria a las Personas Mayores – Montes Claros, MG, Brasil, 2015–2024.

Variables	n	%
Hipertensión arterial sistémica	510	78,8
Diabetes mellitus	241	37,2
Demencia	248	38,3
Depresión	334	51,6
Polifarmacia	280	43,3
Fractura previa	170	26,3
Artritis/Artrosis	302	46,7
Enfermedades del tiroides	108	16,7

Fuente: Elaborado por los autores.

Los resultados de los exámenes de densidad ósea revelaron valores compatibles con osteoporosis en 300 mujeres (46,4 %). Por su parte, se identificó osteopenia en 239 mujeres mayores (36,9 %) y se observó un resultado normal en solo 108 (16,7 %) de las mujeres evaluadas.

La Tabla 3 presenta los resultados de los análisis bivariados. Algunos datos relativos a las variables nivel educativo e IMC no estaban disponibles en el momento de la recopilación y se consideraron omitidos para que no interfirieran en el análisis. Se evaluó la presencia de multicolinealidad entre las variables continuas mediante la matriz de correlación, sin observarse coeficientes superiores a 0,7. Para el modelo final, solo se mantuvieron las variables con p < 0,05.

**Tabla 3:** Análisis bivariado de los factores asociados a la osteoporosis en mujeres mayores atendidas en un Centro de Referencia en Atención Sanitaria a las Personas Mayores – Montes Claros, MG, Brasil, 2015–2024.

Variable	Osteoporosis				Valor p*
	Presente		Ausente		
Nivel educativo (años de estudio)					0,127
≤ 4	242	48,0	262	52,0	
> 4	54	40,6	79	59,4	
Edad (años)					0,001
≥ 80	130	54,6	108	45,4	
< 80	170	41,6	239	58,4	
Índice de masa corporal					<0,001
< 27	182	54,3	153	45,7	
≥ 27	105	36,8	180	63,2	
Tabaquismo					0,999
Anterior/Actual	32	46,4	37	53,6	
No fumador	268	46,4	310	53,6	
Alcoholismo					0,727
Anterior/Actual	31	18,4	33	51,6	
No bebedor	269	46,1	314	53,9	
Consumo de café					0,134
Sí	120	50,2	119	49,8	
No	180	44,1	228	55,9	
Sedentarismo					0,071
Sí	201	44,1	255	55,9	
No	99	51,8	92	48,2	
Polifarmacia					0,411
Sí	135	48,2	145	51,8	
No	165	45,0	202	55,0	
Hipertensión arterial					0,776
Sí	235	46,1	275	53,9	
No	65	47,4	72	52,6	
Diabetes mellitus					0,055
Sí	100	41,5	141	58,5	
No	200	49,3	206	50,7	
Depresión					0,162
Sí	146	43,7	188	56,3	
No	154	49,2	159	50,8	
Demencia					0,870
Sí	116	46,8	132	53,2	
No	184	46,1	215	53,9	
Fractura previa					0,199
Sí	86	50,6	84	49,4	
No	214	44,9	263	55,1	
Artritis/artrosis					0,208
Sí	148	49,0	154	51,0	
No	152	44,1	193	55,9	
Enfermedades del tiroides					0,407
Sí	54	50,0	54	50,0	
No	246	45,6	293	54,4	

Fuente: Elaborado por los autores.

A partir de estos análisis, las variables: nivel educativo, edad, IMC, consumo de café, sedentarismo, diabetes mellitus, depresión y antecedentes de fractura se evaluaron conjuntamente mediante regresión logística binaria. Tras el análisis múltiple, las variables que permanecieron asociadas a la osteoporosis fueron: edad igual o superior a 80 años (p = 0,006; OR = 1,60; IC del 95 %: 1,14–2,24) e IMC < 27 (p < 0,001; OR = 1,97; IC del 95 %: 1,42–2,74). La prueba de Hosmer-Lemeshow indicó un buen ajuste del modelo en todas las etapas (p > 0,05), lo que sugiere que las predicciones no difieren significativamente de los valores observados.

## DISCUSIÓN

La osteopenia y la osteoporosis representaron afecciones clínicas con una elevada prevalencia en la población estudiada. Estos datos concuerdan con otros estudios que han demostrado una elevada prevalencia de osteoporosis en la población femenina. Además, la literatura científica registra un aumento progresivo de la pérdida ósea en mujeres mayores de 50 años, llegando a afectar a dos tercios de la población femenina a los 90 años^(16)^. Aunque este no es un estudio poblacional, los resultados similares resaltan la magnitud del problema. La osteoporosis, una enfermedad silenciosa que afecta especialmente a las mujeres después de la menopausia, es la principal responsable de las fracturas en personas mayores de 50 años. Además, se asocia con un aumento significativo de la morbilidad y mortalidad entre los pacientes con dicha afección clínica^(17)^.

En un estudio realizado en una clínica especializada en el sur de Brasil, los investigadores descubrieron que el 49,8 % de las participantes presentaban osteopenia y el 13,7 % tenían osteoporosis. Por lo tanto, los resultados son similares a los encontrados en el presente estudio y las diferencias observadas pueden ser un reflejo de la población más joven evaluada^(9)^.

Además, una investigación realizada en la India, con mujeres de 50 a 80 años que se sometieron a un examen de densitometría, registró que el 37,5 % presentaba osteoporosis y el 44,7 % presentaba osteopenia, lo que indica un elevado porcentaje de mujeres con compromiso de la calcificación ósea^(15)^.

Cabe destacar que los estudios citados se realizaron a partir de mujeres evaluadas en clínicas de densitometría. Teniendo en cuenta la población general, un estudio estadounidense registró que, en 2017–2018, la prevalencia ajustada por edad de la osteoporosis entre las mujeres de 50 años o más fue del 19,6 %, mientras que la prevalencia de la osteopenia en el mismo grupo de edad fue del 51,5 %. En comparación con años anteriores, los autores observaron un aumento de la prevalencia de la afección en la población general^(18)^. En el contexto de América Latina, la literatura señala que la osteoporosis es una afección de salud global que afecta entre el 30 % y el 50 % de las personas mayores de 50 años^(19)^.

En el presente estudio, tras un análisis múltiple, se identificaron solo dos variables asociadas de manera independiente con el desarrollo de la desmineralización ósea, a saber, la edad avanzada, especialmente en el caso de las mujeres de edad avanzada (de 80 años o más), y el índice de masa corporal (IMC) inferior a 27 kg/m^2^. Las demás variables evaluadas no se mantuvieron en el modelo final.

De manera análoga, en relación con la edad, la literatura existente señala que el envejecimiento es un factor de riesgo para el desarrollo de la osteoporosis. El resultado obtenido en la muestra estudiada es consistente con investigaciones que evidenciaron una pérdida progresiva de masa ósea, principalmente entre las mujeres de 80 años o más. Otros estudios epidemiológicos también han demostrado que la prevalencia de complicaciones relacionadas con la osteoporosis aumenta significativamente con la edad, especialmente en lo que respecta a la aparición de fracturas por fragilidad^(20,21)^.

Un IMC inferior a 27 kg/m^2^ se ha asociado con la osteoporosis, y las personas con un IMC por debajo de este umbral tienen mayor probabilidad de presentar la enfermedad en comparación con aquellas con un IMC igual o superior. En un estudio realizado por Martini et al.^(20)^, que consideró el índice de masa corporal (IMC) como variable aislada, se observó que las personas con un IMC inferior a 25 kg/m^2^ tenían casi tres veces más probabilidades de sufrir una fractura por fragilidad. Por lo tanto, es importante señalar que este punto de corte difiere ligeramente de los valores tradicionalmente utilizados para definir el sobrepeso en pacientes adultos. Sin embargo, es compatible con los límites de normalidad esperados para la población de edad avanzada, de acuerdo con las directrices aceptadas por el Ministerio de Salud^(22,23)^.

Cabe destacar que un IMC excesivo se asocia clásicamente con diversos riesgos para la salud, incluyendo el desarrollo de diabetes tipo 2, enfermedad hepática grasa no alcohólica, enfermedades cardíacas y apnea obstructiva del sueño^(22,23)^. Además, la obesidad puede desencadenar una respuesta inflamatoria crónica, lo que también puede aumentar la posibilidad de osteoporosis y fracturas por fragilidad. De este modo, un IMC adecuado, pero no excesivo, puede permitir que las personas mayores tengan una mejor densidad mineral ósea, equilibrando los beneficios para la salud ósea con la prevención de otros problemas de salud asociados a la obesidad^(22,24)^.

Además de los factores mencionados como asociados a la presencia de osteoporosis, es importante destacar que algunas variables señaladas en otros estudios no se mostraron asociadas a la disminución de la densidad mineral ósea de las personas mayores evaluadas. No hubo resultados que respaldaran, por ejemplo, la asociación entre algunos hábitos de vida como el sedentarismo, el tabaquismo y el alcoholismo con la aparición de osteoporosis. Sin embargo, cabe señalar que es posible que esta falta de asociación se deba a un registro inadecuado en los expedientes médicos en relación con dichas variables.

Un estudio realizado en Picos (Piauí, Brasil), a pesar de no haber presentado una asociación directa entre el alcoholismo y la osteoporosis, mostró que el consumo de bebidas alcohólicas se asoció con un mayor riesgo de fracturas en la población estudiada. Los investigadores del estudio sugieren que el consumo excesivo de alcohol puede estar relacionado con la reducción de la densidad mineral ósea y de la calidad del hueso, como resultado del estrés oxidativo causado por la sustancia. Además, la intoxicación aguda por alcohol tiene el potencial de aumentar el riesgo de caídas en esta población^(25)^. Cabe destacar que las muestras evaluadas, tanto en este estudio como en el realizado en Picos (PI), están formadas exclusiva o predominantemente por mujeres, entre las cuales hay un bajo informe de consumo de alcohol, lo que puede haber contribuido a los resultados encontrados.

Además, una investigación italiana reveló una asociación positiva y equivalente entre el tabaquismo activo y pasivo en la susceptibilidad a la aparición de osteoporosis^(26)^. En el presente estudio, no se identificó esta relación. Sin embargo, cabe destacar que, al igual que con el alcoholismo, la proporción de mujeres que informaron consumo de tabaco es considerablemente pequeña.

Por su parte, la asociación entre la práctica de actividad física, evaluada mediante el registro de caminatas de más de una hora, y la aparición de osteoporosis se señaló en un estudio chino reciente^(27)^. Esta asociación se reiteró en otro estudio que evaluó la relación entre la osteoporosis y los estilos de vida saludables en personas mayores. Se destacó positivamente la actividad física, mientras que el abuso del alcohol y el tabaquismo mostraron impactos negativos sobre la calidad ósea^(28)^.

Los resultados no establecieron una asociación entre el consumo de café y la reducción de la densidad mineral ósea en la población evaluada. Aunque es una variable registrada en algunos estudios, existe una falta de claridad en la literatura sobre la relación entre la osteoporosis y el consumo de café y té^(27)^. Sobre esta cuestión, un estudio de revisión sistemática concluyó que puede existir una relación dependiente de la dosis entre el consumo de café, la osteoporosis y la incidencia de fracturas de cadera. Los autores destacan que la definición de consumo alto o bajo de café varía entre los estudios y señalan que se necesitan nuevas investigaciones con diseños específicos para confirmar los efectos independientes del consumo de café en la salud ósea^(29)^.

La asociación entre la osteoporosis y la polifarmacia, registrada en una investigación realizada en el norte del país^(30)^, no se observó en este estudio. Esta situación refleja, probablemente, alguna particularidad regional, pero es posible que la forma de asignación de los pacientes al estudio también justifique la divergencia observada. Existe la posibilidad de que la polifarmacia sea un indicador de otras condiciones inherentes al proceso de envejecimiento, con un aumento de las comorbilidades, incluida la osteoporosis.

Aunque la realización del estudio en un centro de referencia en salud del anciano pueda representar una limitación en cuanto a la generalización de los resultados, cabe destacar que la elección del lugar ofreció la oportunidad de acceder a los datos de los expedientes médicos y a los resultados de los exámenes recopilados y realizados por profesionales calificados, lo que permitió una mayor precisión y calidad de los hallazgos. Además, la muestra estudiada es representativa, dado que se trata de pacientes derivadas por los servicios de Atención Primaria, lo cual fue un criterio definido previamente para la inclusión de las pacientes en el estudio. Al tratarse de una investigación transversal, no es posible establecer relaciones de causalidad entre las variables analizadas. Además, la dependencia de la información registrada en los expedientes médicos provocó la pérdida de algunos datos, lo que puede representar un sesgo de información, minimizado mediante el análisis estadístico.

Aunque el análisis de los expedientes médicos mostró que la mayoría de las pacientes hacía un uso irregular de los bifosfonatos, especialmente del alendronato, este hallazgo no fue objeto de investigación en el presente estudio. No se evaluaron aspectos del tratamiento de la osteoporosis, tales como: la adherencia terapéutica, la comprensión de las pacientes sobre la enfermedad y los factores asociados al uso inadecuado de la medicación, lo que representa una limitación de este estudio y pone de manifiesto una laguna de conocimiento que debe explorarse en futuras investigaciones.

## CONCLUSIÓN

Por último, la investigación mostró una elevada prevalencia de osteopenia y osteoporosis en la población evaluada. Se observó, además, que la edad avanzada y el bajo índice de masa corporal (IMC) representan factores de riesgo significativos para el desarrollo de la osteoporosis en las mujeres. Estos hallazgos sugieren la necesidad de una vigilancia más rigurosa e intervenciones preventivas dirigidas a las personas mayores, especialmente a aquellas con bajo peso corporal. Por lo tanto, las políticas públicas relacionadas con el cuidado de las personas mayores, así como los profesionales de la salud, deben actuar prioritariamente en la implementación de acciones de promoción y prevención en salud, incluyendo la práctica regular de actividad física y la adopción de una dieta equilibrada para alcanzar un IMC adecuado y, por consiguiente, contribuir a la reducción de la incidencia de la osteoporosis y sus complicaciones. Además, se necesitan más estudios longitudinales para comprender más a fondo los mecanismos relacionados con estas asociaciones.

## Data Availability

El conjunto de datos que respaldan las conclusiones de este estudio está disponible previa solicitud al autor correspondiente.
